# Optimization Protocol of Fixation Method for Trophoblast Retrieval from the Cervix (TRIC): A Preliminary Study

**DOI:** 10.3390/diagnostics10050300

**Published:** 2020-05-14

**Authors:** Min Jin Lee, Soo Hyun Kim, Sung Han Shim, Hee Yeon Jang, Hee Jin Park, Dong Hyun Cha

**Affiliations:** 1 Department of Obstetrics and Gynecology, CHA Ilsan Medical Center, CHA University, Gyenoggi-do KS009, Korea; lydia83@chamc.co.kr; 2 Department of Obstetrics and Gynecology, CHA Gangnam Medical Center, CHA University, Seoul KS013, Korea; soohyunkim@chamc.co.kr; 3 Genetic Laboratory, Fertility Center of CHA Gangnam Medical Center, CHA University, Seoul KS013, Korea; shshim@cha.ac.kr (S.H.S.); jhyeon@chamc.co.kr (H.Y.J.)

**Keywords:** fixation, anti-HLA-G antibody, NIPT (noninvasive prenatal testing), noninvasive, trophoblast, TRIC (trophoblast retrieval and isolation from the cervix)

## Abstract

Extravillous trophoblast cells (EVTs) secreted by the uterine cavity may help overcome limitations associated with prenatal testing currently in use. EVTs are isolated using a routine safe liquid-based Pap test (called ThinPrep); however, the ThinPrep solution contains alcohol that hinders the isolation of intact EVTs. We compared the trophoblastic cell isolation efficiency of two different methods of fixation: Thinprep (pre-fixation method) and formalin (post-fixation method). We analyzed EVTs from 20 pregnant women (5–20 weeks of gestation) who underwent invasive prenatal testing. The percentages of placental β-human chorionic gonadotropin (β-hCG)-expressing cells were calculated. The presence of XY chromosomes were used to confirm pure trophoblast cells by fluorescence in situ hybridization. The β-hCG-positive cells obtained from pre- and post-fixation were 66.4 ± 13.3% and 83.2 ± 8.1% (*p* = 0.003), respectively, and fluorescence-positive cells were 11.1 ± 2.1% and 23.8 ± 4.8%, respectively (*p* = 0.001). Post-fixation was found to be more efficient in isolating non-trophoblast cells than pre-fixation. For the successful clinical application of trophoblast retrieval and isolation from the cervix in prenatal genetic testing, each step should be optimized for consistent and reliable results.

## 1. Introduction

The current average age of mothers at childbirth is over 30 years in developed countries due to women pursuing higher education and developing their careers, women marrying at an older age, and advancements in assisted reproductive technology [1]. The birth rate for women aged 35–44 years steadily increased from 5.2% to 15.5% in 2014 [2]. Emphasis should be put on the importance of prenatal genetic testing because the risk of fetal chromosomal abnormalities increases in childbearing women of advanced maternal age of >35 years [3].

Confirmatory prenatal genetic tests, such as chorionic villus sampling (CVS) and amniocentesis, are invasive procedures. These tests are associated with a procedure-related miscarriage rate of 0.1–2% that increases maternal anxiety [4,5]. Thus, patients and healthcare providers require noninvasive and safe tests that can be performed early during pregnancy. Many studies are currently underway to help address these issues. The first genetic test, wherein Y chromosomes were sequenced from cell-free fetal DNA (cffDNA) extracted from maternal blood, was performed in 1997 [6]. Given the availability and benefits of noninvasive prenatal testing as a screening method, it has gained popularity among pregnant women seeking to avoid the risk of procedure-related miscarriage [7]. Moreover, the prenatal testing of cffDNA in maternal blood can be performed earlier (9–10 weeks’ gestation) than invasive tests (CVS: 10–13 weeks; amniocentesis: 15–16 weeks) [8]. However, using maternal blood in screening includes a small fraction of fetal cells (4–10%) and fragmented DNA (146 base pairs) [9]. cffDNA testing is currently limited to the prenatal assessment of trisomy 13, 18, 21, and sex chromosomal anomalies, with limited positive predictive values for additional genetic conditions [10]. cffDNA is not recommended for the genetic evaluation of the etiology of ultrasound anomalies, as both resolution and sensitivity, or negative predictive values, are inferior to those of invasive tests [10]. To overcome this, some investigators are studying cervical trophoblasts.

Transcervical trophoblasts were discovered in 1971, and extravillous trophoblast cells (EVTs) have consistently been isolated by various methods, such as aspiration, endocervical canal or uterine cavity lavage, and cytobrushing [11,12]. Investigators have attempted to determine the ideal sampling method. The best method should be cost effective, devoid of infectious or fatal complications, and simple to perform, and it should not affect the pregnancy. Sampling using a cytobrush satisfies all these criteria, and, hence, it has been used in most trophoblast retrieval and isolation from the cervix (TRIC) studies since 2009 [13,14]. The TRIC protocol has evolved since then; moreover, fetal genome profiling using single-cell approaches has been reported [9,15]. Because TRIC is available from five weeks gestation, genetic defects can be identified before the end of the embryonic period, and this early diagnostic timing enables speculation on the application of in-utero gene therapy [9]. Nevertheless, TRIC is not yet available in routine obstetric practice since the success rates of obtaining trophoblast cells vary, and post-sampling processes need standardization. Furthermore, its efficiency has not been reported [14].

Thus, TRIC requires standardization and optimization for successful clinical application. Our preliminary results showed that the duration of exposure to a fixing solution is very important. TRIC can be used to fix EVTs by using an alcohol-based ThinPrep solution. Alcohol usually hampers the isolation of intact trophoblast cells. Thus, we used a fixing solution after the washing steps for high yields of intact trophoblast cells. The purpose of this study was to compare the trophoblastic cell isolation efficiency of two different methods of fixation: ThinPrep (pre-fixation method) and formalin (post-fixation method).

## 2. Materials and Methods

### 2.1. Patient Selection

**Figure 1:**
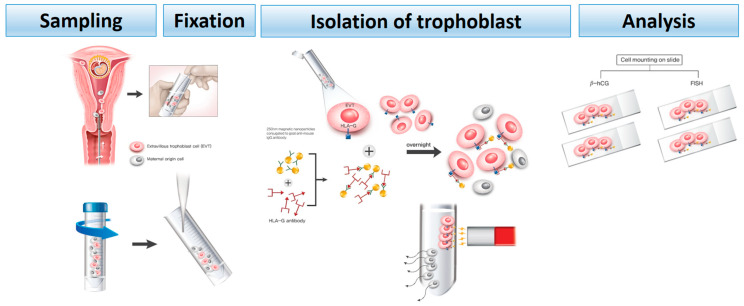
Four-step methodology for post-fixation trophoblast retrieval and isolation from the cervix (TRIC). Sampling: A cytobrush was used as it is used in a Pap test. Fixation: The collected sample was immersed in phosphate-buffered saline (PBS) and transferred to the laboratory, immediately followed by the removal of mucus using 3% acetic acid (300 µL/10 mL) at room temperature for 5 min. It was then fixed with 3.7% formalin for 10 min at 4 °C. Isolation: Magnetic nanoparticles (250 nm) conjugated to goat anti-mouse immunoglobulin G (IgG) antibody were allowed to bind the antibody against human leukocyte antigen G (HLA-G) (specifically expressed in trophoblast cells), and the trophoblast cells were precipitated using a magnet. The bound cells attached to the magnetic stand were the trophoblast cells, and the non-bound cells were the maternal cells. Analysis: Four slides were prepared using Cytospin to confirm that the isolated cells were trophoblast cells using β-human chorionic gonadotropin (β-hCG) and fluorescence in situ hybridization (FISH).

The Institutional Review Board of Gangnam Cha Medical Center (GCI-17-38) approved this study (9 September 2018), and all participating women provided written informed consent. This study included a total of 20 patients who visited the Gangnam Cha Medical Center between 1 November 2018 and 31 April 2019. The inclusion criteria were normal, intrauterine, and singleton pregnancy; gestational age within 5–20 weeks; and those who were scheduled to undergo a Papanicolaou (Pap) test. Patients with multiple pregnancies and active vaginal bleeding were excluded. We analyzed 20 samples that were used to identify fetal sex chromosomes by invasive prenatal testing, such as CVS and amniocentesis. These tests were performed because the mothers were of advanced maternal age with abnormal ultrasound, positive maternal biochemical serum marker, and a family history of chromosome aneuploidy. A Pap test was performed before the invasive tests in all consenting pregnant women. These samples underwent TRIC using a basic four-step process (Figure 1). The protocol is described in subsequent subsections.

### 2.2. Endocervical Sampling

The patient was put in the lithotomy position and a cytobrush was inserted into the external os up to 2 cm and fully rotated by 360° to obtain sufficient cell mass. To compare two different fixation methods, the samples were quickly immersed in the ThinPrep^®^ PreservCyt^®^ solution (Cytyc Corporation, Marlborough, MA, USA) or phosphate-buffered saline (PBS). ThinPrep solution’s chemical composition are methanol (30–60%) and water (40–70%). The immersion of the endocervical samples was performed as described previously [9,12,15,16,17,18]. Briefly, the endocervical samples in ThinPrep^®^ PreservCyt^®^ were immediately fixed (including mucus). The cells were counted and stored at 4 °C.

In the other fixation protocol, the samples were quickly immersed in PBS and immediately transferred to the laboratory. To remove the mucus, samples were treated with 3% acetic acid (300 µL/10 mL) at room temperature for 5 min. After centrifugation at 900× *g* for 5 min at 4 °C, the cells were washed three times with cold PBS. Subsequently, the cells were fixed using 3.7% formalin for 10 min at 4 °C. Fixed cells were centrifuged at 900× *g* for 5 min, washed three times with cold PBS, counted, and immediately stored at 4 °C.

Because the ThinPrep contained a fixative solution, it was immediately fixed with trophoblast cells and other maternal cells together. It was defined as a pre-fixation method in this study because the fixation was performed before the maternal cells were removed. On the other hand, the method using formalin was defined as post-fixation because the trophoblast was fixed after the maternal cells were removed to some extent after sampling.

### 2.3. Immunomagnetic Isolation of Trophoblast Cells

The mouse anti-human leukocyte antigen G (HLA-G) antibody (10 µg/mL, Clone 4H84, BD Biosciences, Pharminge, CA, USA) was incubated with 20 μL of 250 nm magnetic nanoparticles conjugated to a goat anti-mouse immunoglobulin G (IgG) antibody (Clemente Associates, Madison, CT, USA) overnight at 4 °C. The next day, the non-bound nanoparticles were then washed three times with cold PBS in a magnetic strand. Then, the endocervical cells were resuspended in 1.5 mL PBS containing 1% bovine serum albumin (BSA) and anti-HLA-G antibody-coupled nanoparticles, and then the cells were incubated overnight at 4 °C with mixing. The bound cells and non-bound cells were collected in each tube after magnetic immobilization. The bound cells were washed three times with cold PBS in a magnetic strand. Ultimately, the bound cells attached to the magnetic strand were considered trophoblast cells, and the non-bound cells were considered the maternal cells.

### 2.4. Immunohistochemistry

For immunofluorescence microscopy, the isolated anti-HLA-G antibody-positive cells and anti-HLA-G antibody-depleted cells were suspended in 200 µL of PBS on a slide and centrifuged at 1500 rpm for 5 min using the Cytospin 7620 (Wescor Inc., Logan, UT, USA). Cells attached to the slides were dried and blocked in PBS with 3% BSA at 4 °C for >1 h. The slides were incubated with β-hCG primary antibody (10 μg/mL, 5H4-E2, Thermo Scientific, Waltham, MA, USA) overnight at 4 °C and washed three times using PBS containing 0.5% Tween 20 for 10 min at room temperature. Subsequently, the slide was incubated with Alexa Fluor^®^ 555 goat anti-mouse IgG (5 μg/mL, Invitrogen Carlsbad, CA, USA) at 4 °C for 1 h and washed three times with PBS. The cells were then stained with 4′,6-diamidino-2-phenylindole dihydrochloride (1 μg/mL) at room temperature for 10 min and washed three times with PBS. The cells were mounted on slides with a coverslip and observed under the Axio Imager 2 fluorescence microscope (Carl Zeiss, Thornwood, NY, USA). The percentage of cells expressing β-hCG was calculated.

### 2.5. Fluorescence in Situ Hybridization (FISH)

Isolated cells were incubated with fluorescence in situ hybridization (FISH) probes against chromosomes X and Y: DXZ1 Alpha Satellite SpectrumOrange and DYZ1 satellite III SpectrumGreen were the X and Y chromosome probes (Abbott Molecular, IL, USA), respectively. FISH was performed according to the manual, and signals were analyzed using the Axio Imager A2 fluorescence microscope (Carl Zeiss, Thorwood, NY, USA) with the Isis FISH imaging system.

### 2.6. Statistical Analysis

All statistical analyses were performed using SPSS version 18.0 (SPSS, Chicago, IL, USA). Data are expressed as mean ± standard deviation, and the pre-fixation group was compared with the post-fixation group using the Mann–Whitney *U* test (for non-normal distribution). *p*-values < 0.05 were considered statistically significant. This was an explorative study, thus justifying the low sample size and the absence of sample size calculation.

## 3. Results

We compared the two fixation methods for isolating trophoblast cells from 10 endocervical samples each. The mean age of the pregnant women was 32.9 ± 4.1 and 34.9 ± 5.4 years in the pre- and post-fixation groups, respectively (*p* = 0.356). The total number of endocervical cells obtained from 20 endocervical samples ranged between 1.4 × 10^5^ and 3.4 × 10^6^ and was independent of the gestational age (*p* = 0.554) (Table 1 and Table 2).

**Figure 2:**
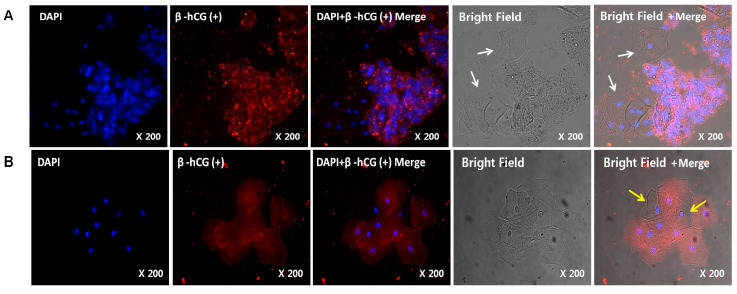
Expression of β-hCG in cells isolated using HLA-G coupled to magnetic nanoparticles after pre-fixation (**A**) with an alcohol-based fixer and post-fixation (**B**) using formalin. Nuclei counterstained with 4′,6-diamidino-2-phenylindole dihydrochloride (DAPI: blue) are shown in the left panel, and images of the secondary antibody conjugated with Alexa Fluor^®^ 555 (red) are shown in the second panel. DAPI- and β-hCG-stained images were merged, as shown in the third panel. DAPI-stained, β-hCG-stained, and bright-field images were merged, as shown in the right panel. Cell aggregation was observed with the pre-fixation protocol using bright-field microscopy (**A**). The fetal or maternal origin of these cells was impossible to distinguish. Ambiguous cells were classified as non-trophoblast cells (white arrows). Compared to (**A**), cell aggregation remarkably improved upon post-fixation (**A**). Moreover, non-trophoblast cells (yellow arrows) were clearly visible.

**Figure 3:**
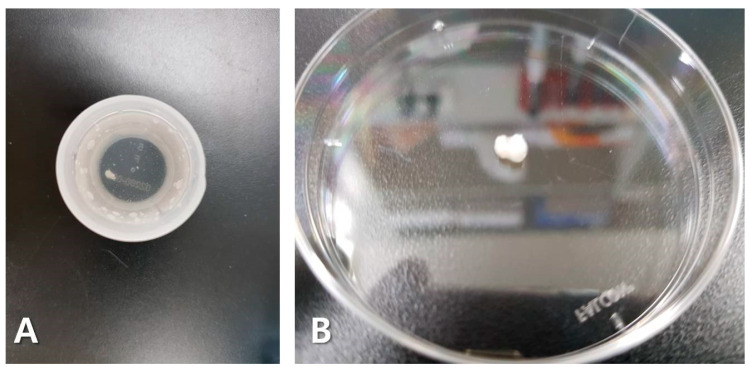
Macroscopically aggregated tissues. As soon as the sample was dipped in the alcohol-based solution, the cells aggregated (**A**), and when one of them was taken out, we could observe hardened aggregated cells (**B**).

The effective removal of non-trophoblast cells was identified as follows: The mean values of the anti-HLA-G-positive cells from isolated trophoblasts were 2672.2 and 1141.1 in the pre- and post-fixation groups, respectively (*p* = 0.043). We compared the pre- and post-fixation methods by labeling immunomagnetically isolated trophoblast cells with β-hCG. As shown in Figure 2A, there was residual mucus after pre-fixation that aggregated with the trophoblast cells during immunomagnetic separation. We observed that these non-trophoblast cells did not bind to the β-hCG antibody. Whether these cells were of trophoblast or maternal origin could not be distinguished due to their aggregated nature. In contrast, the aggregated cells could not be detected in the post-fixation method (Figure 2B). The β-hCG-expressing and non-expressing cells were easily distinguishable from the trophoblast and maternal cells, respectively (Figure 3). Most importantly, the residual mucus and cell aggregation observed in the pre-fixation method were minimized in the post-fixation method. Moreover, immunofluorescence microscopy using a bright-field halogen lamp helped differentiate between the trophoblast and non-trophoblast cells. Thus, 66.4 ± 13.3% and 83.2 ± 8.1% β-hCG-positive trophoblast cells were obtained using the pre- and post-fixation methods, respectively (Table 2). There were significant differences between the two fixation methods (*p* = 0.003).

**Figure 4:**
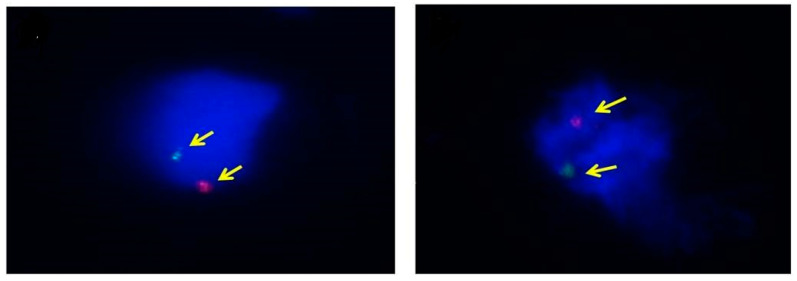
Trophoblast cells from TRIC samples analyzed by FISH. The yellow arrows point to the chromosomes. Male fetuses were labeled with DXZ1 Alpha Satellite SpectrumOrange and DYZ1 satellite III SpectrumGreen probes against the X (red) and Y (green) chromosomes, respectively. Nuclear chromatin was labeled with DAPI (blue).

Moreover, FISH detected the presence of X and Y chromosomes in samples identified by invasive tests, such as CVS or amniocentesis, to confirm the purity of trophoblast cells (Figure 4). FISH revealed positive rates of 11.1 ± 2.1% (8.6%–13.5%) and 23.8 ± 4.8% (19.4%–29.4%) after pre- and post-fixation, respectively (*p* = 0.001) (Table 3).

## 4. Discussion

### 4.1. Main Findings

Trophoblast cells develop during the placentation period (5–12 weeks after the implantation of the conceptus). During decidualization, which is the transformation of proliferating endometrial stromal cells into specialized secretory cells, EVTs invade the uterine blood vessels (endovascular EVTs) and glands (endoglandular EVTs). In the lateral placental margin, some of the endoglandular EVTs penetrate the uterine cavity and reach the cervix after transport. This is the general hypothesis that forms the basis of TRIC experiments [14]. The fundamentals of sampling, storage, isolation and DNA extraction of TRIC have been established since 1971 [9]. However, the reproducibility of TRIC remains controversial due to its 75%–100% success rates in confirming fetal tissues using β-hCG (specifically expressed in the placenta) [14]. This limits the clinical application of TRIC as a prenatal diagnostic tool. Therefore, it is imperative to establish a reproducible and accurate method that achieves consistent results in different patients [13,19,20].

Most TRIC studies use an alcohol-based ThinPrep solution. This is considered advantageous since it can be performed alongside routine Pap tests in early pregnancy. Based on our findings, this step can be more appropriately referred to as fixation. As soon as the tissue sample was immersed in the solution, the cells macroscopically aggregated during fixation (which occurred rapidly in the presence of alcohol) (Figure 3). Upon microscopic analysis, we found that this phenomenon was so severe that some of the samples could no longer be tested (Figure 2A). In our study, depending on the fixation solution, the two methods were compared based on whether trophoblast cells were fixed together with maternal cells (pre-fixation method) or fixed after maternal cells were removed (post-fixation method). Fixing tissues is an essential aspect in reliably performing a histological examination and determining the success rate of experiments. The most commonly used fixers are glutaraldehyde, formaldehyde, and methanol/acetone. Since formaldehyde is the best fixer in most immunofluorescence methods, we tested the others and used PBS and formaldehyde in post-fixation [21,22]. Post-fixation exhibited less cell aggregation and increased the efficiency of trophoblast cell isolations (Figure 2B). This suggested that, although pre-fixation is convenient, post-fixation increases the success rate of experiments.

Microscopically identifying trophoblast and non-trophoblast cells was difficult. We considered cells expressing β-hCG with a stained nucleus to be trophoblast cells. Ambiguous cells were also observed during this process. Other studies also found obscure cells, but they were either not mentioned or termed resident cells of unknown origin, thus, making it impossible to discern whether these cells were counted as trophoblast or non-trophoblast cells. We used bright-field microscopy to clearly differentiate the two cell types (Figure 2). To the best of our knowledge, this is the first study to use this technique in TRIC research. We believe that the simplicity of this approach will significantly contribute to improving accuracy in calculating the purity of trophoblast cells in future TRIC studies.

TRIC research depends on the efficacy of trophoblast cell isolation. Previous studies were based on the separation of trophoblast cells by anti-HLA-G and magnets. FISH was used to confirm the purity of the trophoblast cells; the FISH and immunofluorescence (using β-hCG) results did not tally. FISH (for pure trophoblast cells with the Y chromosome) confirmed that only 25% of the isolate comprised pure trophoblast cells, while β-hCG staining showed >80% to be pure trophoblast cells. It can be concluded that, unlike the analyses of previous studies, cells isolated from the magnet contained pure trophoblast cells and non-trophoblast cells. Thus, due to this heterogeneity, the purity of trophoblast cells was not 100%, even after post-fixation. Therefore, to use TRIC clinically, it is imperative to remove the non-trophoblast cells through a sequential step down process during cell isolation after fixation; thus, we are actively pursuing this in our laboratory.

### 4.2. Strengths and Limitations

The strengths of this research include the fact that it is the first study to compare the conventional fixing method of TRIC and a novel approach to minimize the ambiguity of cell detection in order to increase the reproducibility of the research. This study significantly contributes to establishing the clinical use of TRIC. On the other hand, the limitations of this study include its small sample size, the broad range of gestational age, and the PBS used in post-fixation that was not immediately delivered to the laboratory. Since a ThinPrep solution is immediately fixed after taking the sample, it is possible to deliver the sample after the outpatient treatment is complete. However, since the sample using PBS is unstable because it is not fixed, it must be immediately delivered to the laboratory. This may limit its clinical application because it reduces the efficiency of the sample transfer.

TRIC is very interesting because complete fetal DNA can be identified using a noninvasive test that can be performed within five weeks of gestation [9,16]. Since confirmatory noninvasive testing is required in early pregnancy, we believe that our findings will help optimize each step of TRIC and lead to a paradigm shift in noninvasive prenatal genetic testing.

## 5. Conclusions

We have found that trophoblast cells can be more efficiently isolated by minimizing cell aggregation through a post-fixation method using PBS instead of an alcohol-based ThinPrep solution. Future studies will need to focus on the feasibility of the method because post-fixation requires the immediate transfer of cells to the laboratory for processing and on clearly identifying which cells are non-trophoblast cells.

## Figures and Tables

**Table 1:** Sample characteristics.

	Sample ID	Maternal Age	Mean ± SD	*p*-Value	GA (Days)	Mean ± SD	*p*-Value	Gravidity/Parity	BMI (kg/m^2^)
Pre-Fixation (ThinPrep)	1	35	32.9 ± 4.1	0.356	119	71.6 ± 26.3 (63.0)	0.260	2/1	22.6
	2	27			87			1/0	15.9
	3	36			51			3/2	21.7
	4	32			50			2/1	22.2
	5	29			58			1/0	19.8
	6	36			112			2/1	20.4
	7	35			54			2/1	24.6
	8	36			45			1/0	23.0
	9	37			68			1/0	23.5
	10	26			72			1/0	20.7
Post-Fixation (Formalin)	1	34	34.9 ± 5.4		77	85.2 ± 26.0 (89.3)		1/0	20.0
	2	35			54			2/1	25.9
	3	41			47			2/1	25.8
	4	31			55			1/0	17.4
	5	25			86			1/0	21.1
	6	40			112			1/0	17.5
	7	30			96			2/0	26.9
	8	38			112			3/2	20.7
	9	41			117			1/0	23.7
	10	34			96			1/0	19.6

GA: gestational age; SD: standard deviation.

**Table 2:** Trophoblast contents and detection of β-hCG.

	Sample ID	GA (Days)	Number. of Cells							β-hCG Detection		
			Total Endocervical Cells	Fixed Cells	Mean	*p*-Value	HLA-G-Positive Cells	Mean	*p*-Value	Positive (%)	Mean ± SD	*p*-Value
Pre- Fixation (ThinPrep)	1	119	4.60 × 10^5^	2.0 × 10^5^	2.38 × 10^5^	0.64	1000	2672.2	0.043	78.0	66.4 ± 13.3	0.003
	2	87	5.35 × 10^5^	2.0 × 10^5^			4250			76.1		
	3	51	4.60 × 10^5^	2.0 × 10^5^			2550			73.9		
	4	50	2.58 × 10^6^	2.0 × 10^5^			867			78.4		
	5	58	3.25 × 10^5^	5.1 × 10^5^			1000			78.0		
	6	112	5.70 × 10^5^	2.0 × 10^5^			2333			71.7		
	7	54	2.40 × 10^5^	2.0 × 10^5^			6500			45.9		
	8	45	5.89 × 10^5^	2.0 × 10^5^			5750			46.7		
	9	68	1.37 × 10^6^	2.3 × 10^5^			1250			53.8		
	10	72	7.60 × 10^5^	2.4 × 10^5^			1222			61.3		
Post- Fixation (Formalin)	1	77	1.60 × 10^6^	3.2 × 10^5^	2.67 × 10^5^		575	1141.1		96.0	83.2 ± 8.1	
	2	54	3.48 × 10^6^	3.4 × 10^5^			2250			88.0		
	3	47	1.40 × 10^5^	1.4 × 10^5^			100			84.0		
	4	55	3.40 × 10^5^	2.0 × 10^5^			625			84.6		
	5	86	8.70 × 10^5^	1.6 × 10^5^			375			86.7		
	6	112	2.48 × 10^6^	1.8 × 10^5^			1277			88.9		
	7	96	7.10 × 10^5^	2.0 × 10^5^			1889			78.8		
	8	112	5.30 × 10^5^	7.1 × 10^5^			900			81.5		
	9	117	4.20 × 10^5^	2.2 × 10^5^			1670			65.7		
	10	96	4.50 × 10^5^	2.0 × 10^5^			1750			78.0		

GA: gestational age; SD: standard deviation.

**Table 3:** Trophoblast contents and FISH.

	Sample ID	GA (Days)	Karyotype (Genetic Test)	Karyotype (FISH)	Fetal Sex (after Delivery)	Number. of Cells (XY/Total)	Y Detection		
							Rate (%)	Mean ± SD	*p*-Value
Pre- Fixation (ThinPrep)	1	119	XX	XX	Female			11.1 ± 2.1	0.001
	2	87	XY	XY	Male	3/35	8.6		
	3	51	XX	XX	Female				
	4	50	XY	XY	Male	7/52	13.5		
	5	58	XY	XY	Male	5/47	10.6		
	6	112	XX	XX	Female				
	7	54	XX	XX	Female				
	8	45	XX	XX	Female				
	9	68	XX	XX	Female				
	10	72	XY	XY	Male	6/51	11.8		
Post- Fixation (Formalin)	1	77	XY	XY	Male	15/51	29.4	23.8 ± 4.8	
	2	54	XY	XY	Male	12/52	17.8		
	3	47	XX	XX	Female				
	4	55	XX	XX	Female				
	5	86	XX	XX	Female				
	6	112	XY	XY	Male	13/45	28.9		
	7	96	XX	XX	Female				
	8	112	XY	XY	Male	12/50	24.0		
	9	117	XY	XY	Male	8/30	23.3		
	10	96	XY	XY	Male	12/62	19.4		

GA: gestational age. SD: standard deviation.
